# Advances in determining the gross tumor target volume for radiotherapy of brain metastases

**DOI:** 10.3389/fonc.2024.1338225

**Published:** 2024-05-08

**Authors:** Shanshan Du, Guanzhong Gong, Rui Liu, Kangning Meng, Yong Yin

**Affiliations:** ^1^Department of Oncology, Affiliated Hospital of Southwest Medical University, Luzhou, Sichuan, China; ^2^Department of Radiation Oncology Physics and Technology, Shandong Cancer Hospital and Institute, Shandong First Medical University and Shandong Academy of Medical Sciences, Jinan, China

**Keywords:** brain metastases, MRI, GTV, delineation, artificial intelligence

## Abstract

Brain metastases (BMs) are the most prevalent intracranial malignant tumors in adults and are the leading cause of mortality attributed to malignant brain diseases. Radiotherapy (RT) plays a critical role in the treatment of BMs, with local RT techniques such as stereotactic radiosurgery (SRS)/stereotactic body radiotherapy (SBRT) showing remarkable therapeutic effectiveness. The precise determination of gross tumor target volume (GTV) is crucial for ensuring the effectiveness of SRS/SBRT. Multimodal imaging techniques such as CT, MRI, and PET are extensively used for the diagnosis of BMs and GTV determination. With the development of functional imaging and artificial intelligence (AI) technology, there are more innovative ways to determine GTV for BMs, which significantly improve the accuracy and efficiency of the determination. This article provides an overview of the progress in GTV determination for RT in BMs.

## 1 Introduction

Brain metastases (BMs) are the most frequent intracranial malignant tumors in adults; among patients with malignant tumors, the incidence rate of BMs in adults is 10%–30%, and that in children is 6%–10% (1). The leading tumor type resulting in BMs is lung cancer, accounting for approximately 20%, followed by melanoma, breast cancer, etc. (1). Notably, the incidence of BMs surpasses that of primary brain tumors, making them the primary cause of mortality from malignant brain diseases (2).

The treatment methods for BMs include whole-brain radiotherapy (WBRT), stereotactic radiosurgery (SRS)/stereotactic body radiotherapy (SBRT), surgery, and systemic therapy (3). In recent years, an increasing amount of clinical evidence supports the application of local RT techniques such as SRS/SBRT in BMs (4). Accurate determination of the gross tumor target volume (GTV) is crucial for ensuring the efficacy of SRS/SBRT. With the development of advanced imaging technology and information science and technology, the use of multimodal imaging technology and artificial intelligence (AI) to improve the accuracy of GTV determination in BMs has become a popular research direction.

## 2 RT for BMs

Radiotherapy (RT) serves as the primary treatment method for BMs. Objective studies have shown that patients with symptomatic BMs typically have a median overall survival (OS) of just 1 month without treatment. However, treatment with glucocorticosteroids alone, such as dexamethasone, can extend the median OS to 2 months (5). Lagerwaard et al. (6) reported that patients who received different doses of WBRT experienced an extended median OS of 3–6 months and a 1-year survival rate of 10%.

WBRT is the standard treatment for multiple BMs (7). However, WBRT often results in delayed adverse events including leukoencephalopathy, associated cognitive dysfunction, cerebral atrophy, and radionecrosis. Cognitive dysfunction is reported in approximately 10%–20% of patients undergoing WBRT (8). To avoid radiation damage to normal brain tissue, local RT methods such as SRS/SBRT have been developed. Multiple international clinical trials support SRS as the preferred treatment for BM patients with one to four metastases (9–12). The JLGK0901 (4) study revealed that patients with five to 10 BMs achieved comparable treatment results with patients with two to four BMs by receiving SRS treatment; the median OS all reached 10.8 months. Therefore, SRS could be considered an alternative treatment for multiple BMs instead of WBRT.

SRS/SBRT requires precise GTV determination and accurate dose delivery, which are directly related to the therapeutic efficacy and prognosis. The small size of BMs and their unclear enhancement affect lesion detection and visibility of tumor boundaries, which are the key challenges that need to be addressed in GTV determination for BMs.

## 3 Advances in GTV determination of BMs based on multimodal imaging

### 3.1 Computed tomography-based GTV determination in BMs

#### 3.1.1 Single-energy CT-based determination

**Figure 1:**
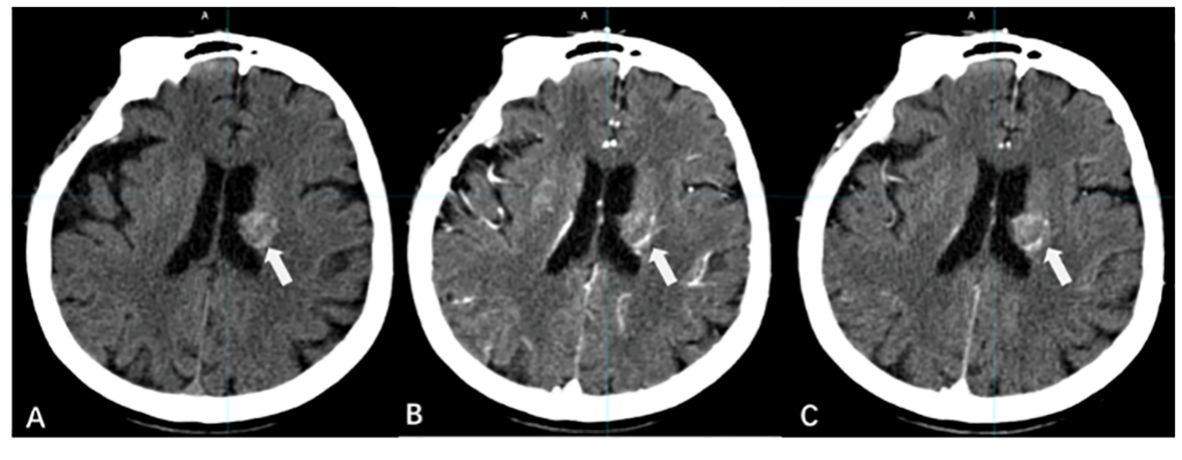
CT imaging manifestations of BMs. [**(A)** CT scan without contrast; **(B)** CT contrast scan in arterial phase; **(C)** CT contrast scan in vein phase].

Computed tomography (CT) simulation images serve as the primary foundation for GTV determination in BMs. CT offers high spatial resolution, minimal distortion, and sensitivity to features such as bleeding, calcification, and structural changes in the surrounding skull of BMs. The linear relationship between the CT value and tissue electron density is the basis for dose calculations in RT planning (12) (**Figure 1**).

However, single-energy CT (SECT) is susceptible to bone artifacts, peri-tumor edematous areas, and fibrosis, limiting its use in GTV determination in BMs. Emerging CT technologies provide a reliable means to improve the precision of GTV determination.

#### 3.1.2 Dual-energy computed tomography-based determination

Dual-energy computed tomography (DECT) improves the signal-noise ratio (SNR) and contrast-noise ratio (CNR) of intracranial abnormal metal deposits, iodine contrast, normal brain tissue, and abnormal lesions, resulting in superior image quality (13). DECT reduces the beam hardening artifacts caused by the skull through a special reconstruction algorithm, improving the image quality of posterior fossa tumors and improving BM imaging (14).

DECT shows greater image enhancement at low tube voltages (15). Karino et al. (16) analyzed energy spectral images of virtual monochromatic images (VMI) with energy levels ranging from 40 to 140 KeV in increments of 1 keV gradient and found that a VMI of 63 KeV significantly improved the overall image quality and BM boundary display. Kraft et al. (17) further compared the differences between 63 keV reconstructed VMI and 120 kV CT in BM imaging and confirmed that the image quality of VMI was significantly better than that of conventional CT, which could improve the reliability and accuracy of GTV determination in BMs.

### 3.2 Multisequence MR-based GTV determination in BMs

Compared with CT, magnetic resonance (MR) provides high soft tissue resolution, clear differentiation between tumors and tissue edema, and avoids radiation injury. MR is thus indispensable for diagnosing, planning treatment, and posttreatment monitoring of brain tumors (18).

Contrast-enhanced MRI (CE-MRI) with gadolinium (Gd)-based contrast serves as the gold standard for identifying BMs (19). However, the contrast of the enhanced region of BMs in CE-MRI is affected by various factors, including the characteristics of the blood–brain barrier (BBB), magnetic field strength, concentration of Gd-based contrast agent (GBCA), relaxivity properties, time elapsed since injection, and the MR imaging technique (20). Derks et al. (12) found that 3T MR was more sensitive than 1.5 T MR in the diagnosis of small-volume BMs with diameters < 5 mm.

Cheng et al. (21) found that 7T MR improved visualization of small structures and subtle brain tumor lesions compared to 3T MR, which had significant potential for the diagnosis, treatment, and monitoring of BMs. However, the increase in magnetic field strength also leads to greater magnetization artifacts, which affects the GTV determination of BMs.

CE-T1WI and T2/Fluid-attenuated inversion recovery (FLAIR) are the most commonly used sequences in MR simulations of BMs (22).

#### 3.2.1 CE-T1WI-based determination

There are differences in the imaging of BMs on T1WI with different imaging bases. A comparative analysis of the detection rates of BMs using enhanced spin-echo (SE) and gradient-echo (GRE) sequences by Suh et al. (23) revealed that, with a layer thickness of 1 mm, 3D SE images had a 20.6% higher detection rate than 3D GRE images. Additionally, for lesions with diameters less than 5 mm, 3D SE images exhibited a 30.1% higher detection rate than 3D GRE images. For the detection of BMs, especially lesions less than 5 mm in size, 3D SE-enhanced images with a 1-mm-layer slice thickness are more suitable.

**Figure 2:**
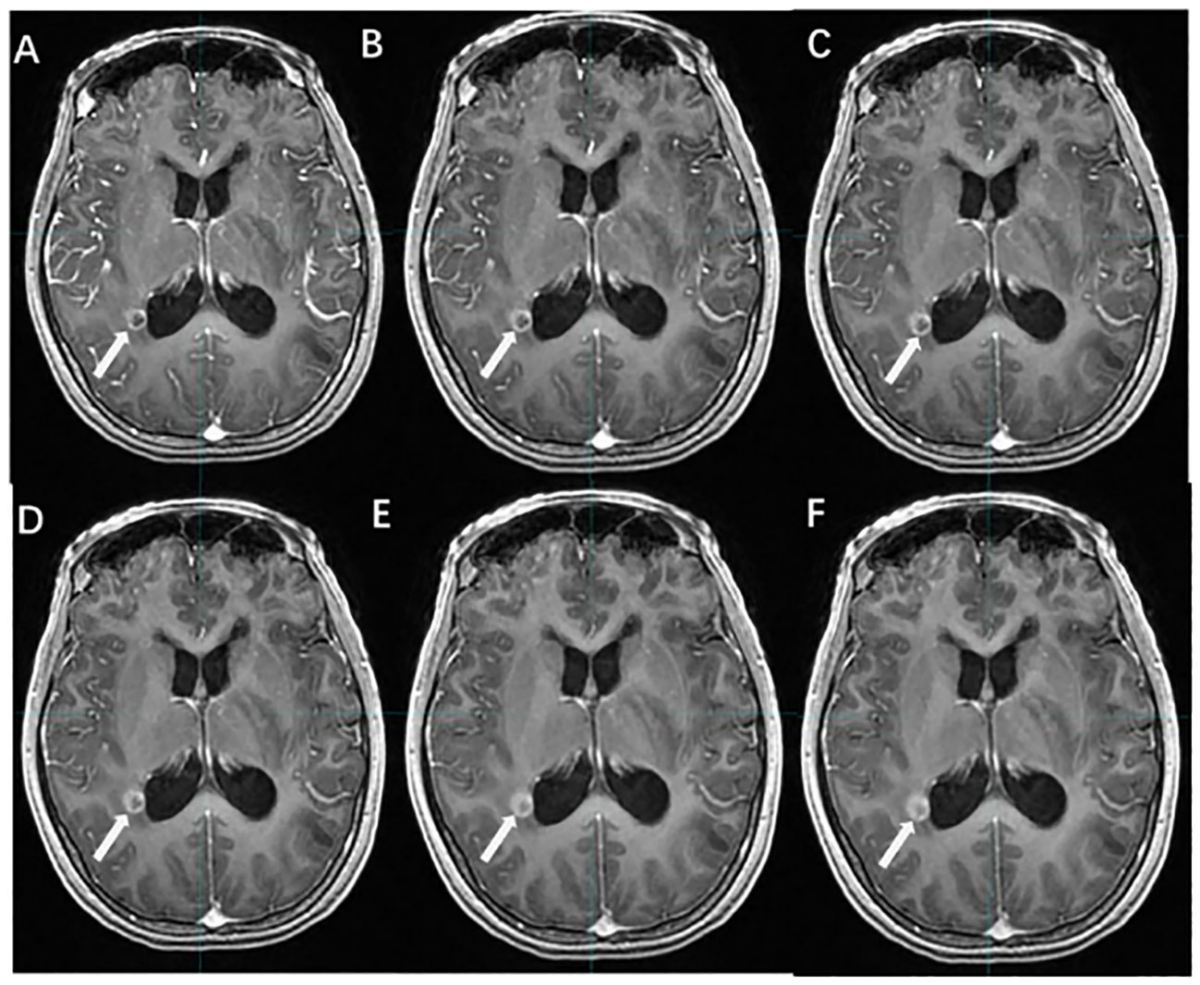
The effect of time-delayed contrast-enhanced T1WI on the visualization of BMs. [**(A–F)** The BM manifest on contrast-enhanced T1WI at 1, 3, 5, 10, 18, and 20 min after Gd-DTPA injection].

Whether delayed enhancement MRI can improve the detection rate of BMs has been controversial (**Figure 2**). Cohen et al. (24) concluded that the detection rate of BMs by delayed CE-MRI is related to lesion volume. For larger BMs, delayed CE-MRI is not more advantageous than immediate postcontrast-enhanced imaging; however, for lesions with diameters ≤ 5 mm, delayed CE-MRI improves the detection rate of lesions and increases the display of tumor boundaries, especially when the delay time is more than 10 min (25–28).

Chen et al. (29) investigated the effect of CE-MRI with different delay times on the generation of small-volume BMs, and the results showed that compared with 10 min after contrast agent injection, the metastatic volumes at 1, 3, and 5 min decreased by 31.6%, 18.5%, and 10.1%, respectively, and the metastatic volumes at 18 and 20 min increased by only 8% and 10%, respectively. Therefore, CE-MRI with a delay time of more than 10 min should be a routine modality for the detection and border display of small-volume BMs.

Several studies have demonstrated that high-dose Gd-based contrast agents (GBCA) improve the demonstration of BMs, and the use of triple-dose contrast agents increases the detection of BMs < 5 mm by 65.6%; however, it also increases the risk of patient complications and the cost of the examination (26, 30–33).

#### 3.2.2 T2/FLAIR-based determination

**Figure 3:**
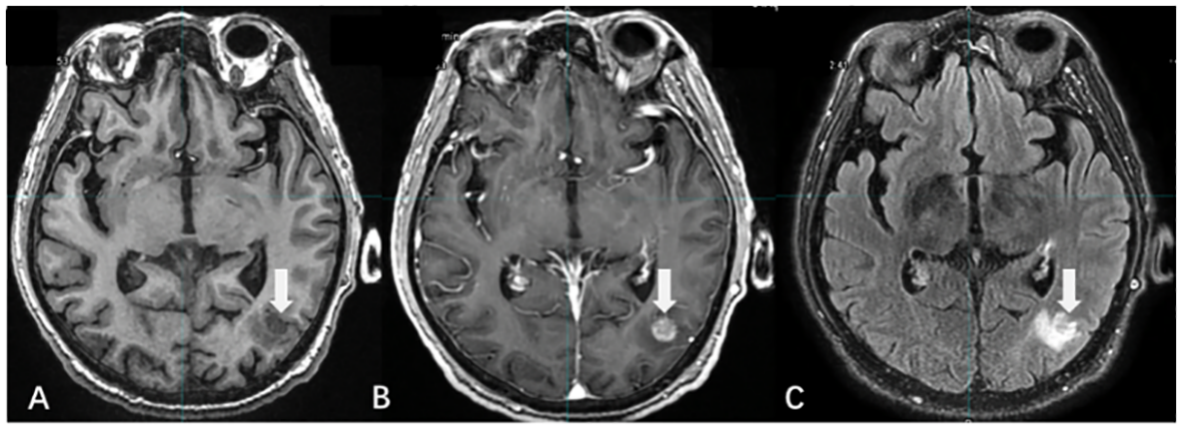
The display differences of BMs among T1WI, T1WI+C, and T2/FLAIR. [**(A)** T1WI without contrast; **(B)** CE-T1WI; **(C)** CE-T2/FLAIR].

Unlike CE-T1WI, T2/FLAIR uses a low concentration of GBCA to enhance the lesion, and the contrast agent required to enhance the lesion is only one-fourth of that required for CE-T1WI (34, 35). Several studies have demonstrated that CE-T2/FLAIR is superior in detecting leptomeningeal metastases, small-volume lesions, and lesions located in superficial areas of the brain (36, 37) (**Figure 3**).

There were differences in the detection rate and enhancement degree of BMs between CE-T2/FLAIR and CE-T1WI. Jin et al. (38) suggested that this was related to the vascular permeability and microvascular density around the lesions of BMs. In lesions with higher density or greater damage to the BBB, the venous leakage of the contrast medium increased, resulting in a large accumulation of GBCA in the extracellular space. The enhancement effect of T2 reduced the degree of enhancement of the lesions and even led to negative enhancement. Thus, the degree of CE-T2/FLAIR enhancement was negatively associated with vascular permeability and demonstrated superior enhancement in BMs with low vascular permeability. CE-T2/FLAIR is an effective supplement to CE-T1WI, and the combined application of the two improves the detection and determination accuracy of BMs (39).

### 3.3 PET-based GTV determination of BMs

Positron emission tomography (PET) is a noninvasive imaging technique used to assess the biological function and metabolism of tumors. 18F fluorodeoxyglucose (18F-FDG) is the most widely used PET tracer, but normal brain tissue also shows high glucose uptake, which affects the GTV determination of BMs (40). Compared to 18F-FDG, the uptake of radiolabeled amino acids is lower, allowing them to cross the intact BBB, revealing BMs beyond CE MRI, and providing new insights for delineating biological targets in BMs (41).

#### 3.3.1 PET/CT

PET/CT is used to obtain information on tumor biology and metabolism, which is valuable for determining biological GTVs. However, it has limitations in detecting BMs, especially for small-volume BMs. Factors such as lower FDG uptake by small BMs and the lower spatial resolution of PET/CT affect image quality, while the high uptake of inflammatory tissue reduces diagnostic specificity (42). In a study of more than 900 patients with BMs from lung cancer, Li et al. (43) found that CE-MRI had a higher sensitivity than FDG PET/CT for diagnosing BMs in patients (77% vs. 21%).

**Figure 4:**
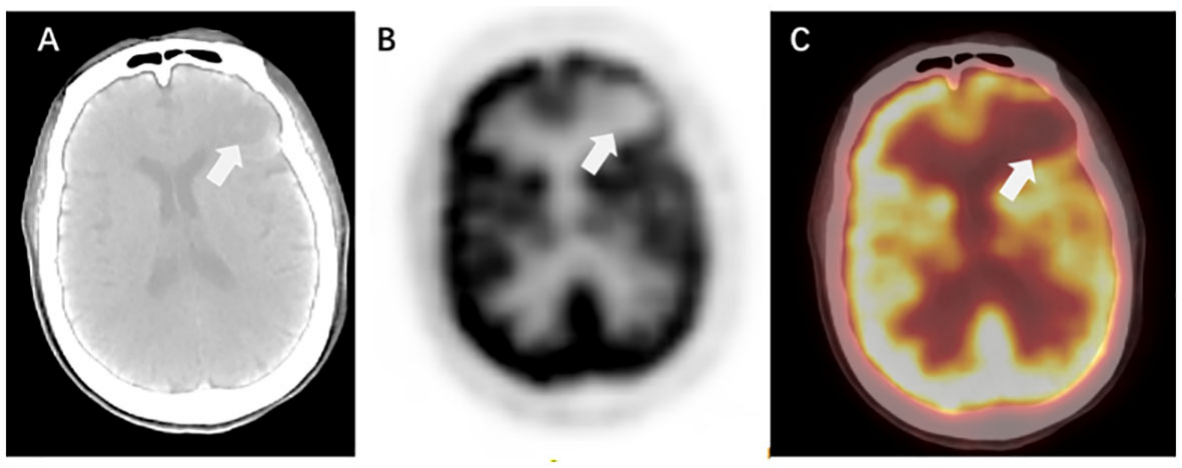
PET/CT manifestations of BMs. [**(A)** CT without contrast; **(B)** PET; **(C)** PET/CT].

PET/CT is primarily used to detect extracranial lesions and is not highly sensitive for BMs, especially those with small volumes. Therefore, PET/CT is often complemented by the combination of enhanced MRI or CT (**Figure 4**).

#### 3.3.2 PET/MR

PET/MRI has the advantage of simultaneously capturing both structural and functional aspects of tumors, which enhances RT planning and the assessment of treatment response (44). By combining high-resolution soft tissue PET with MRI, PET/MR provides a reliable basis for precise GTV determination in BMs.

Singnurkar et al. (45) compared the clinical performance of FDG PET/MR with FDG PET/CT in tumor imaging. FDG PET/MR was found to be similar to FDG PET/CT in detecting local lymph nodes and distant metastases but superior in assessing the local extent of tumors. Similarly, another study by Sekine et al. (46) demonstrated a 6.7% improvement in PET/MR imaging compared to PET/CT for occult tumors, including brain tumors.

### 3.4 CT/MRI fusion

CT provides crucial anatomical information and electron density data for RT planning and dose calculation, but it lacks sufficient soft tissue contrast, making it unreliable to determine GTV only based on CT alone (47). MRI provides high soft tissue resolution, enabling differentiation between active tumors and edematous tissue. The fusion of CT and MRI images combines the advantages of both modalities. However, differences in the underlying principles of CT and MRI imaging modalities, poor reproducibility of body fixation, poor accuracy of patient positioning, interlayer differences in layer thicknesses of images from different modalities, and tumor regression due to treatment all contribute to the poor quality of CT/MRI fusion images, which affects the visualization of the BMs and the accuracy of the GTV determination.

**Figure 5:**
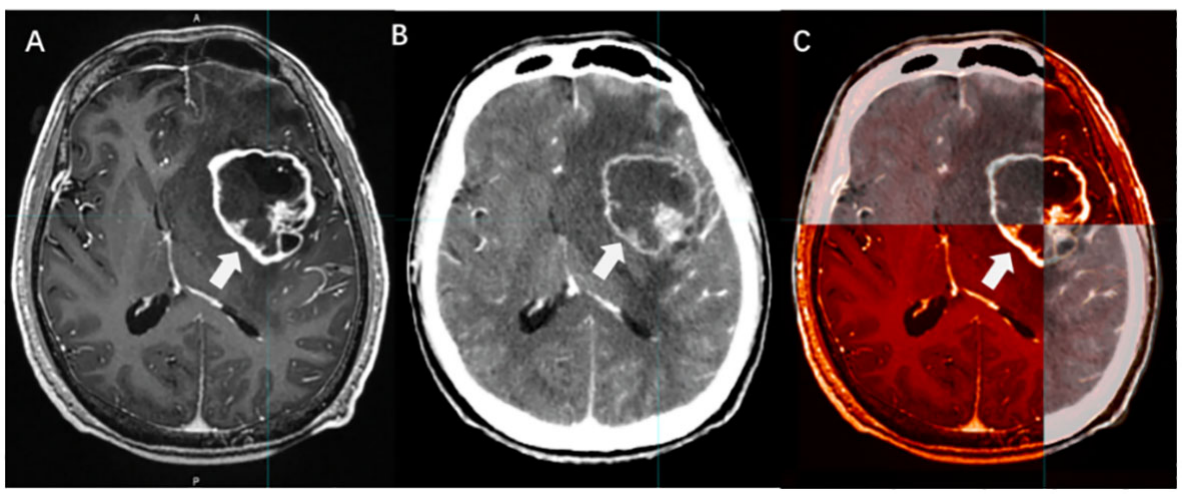
The boundary determination of BMs applying CT/MRI fusion. [**(A)** CE-T1WI images; **(B)** Enhanced CT images; **(C)** CT/MRI fusion].

Few controlled studies of the volume and dosimetric effects of CT/MRI fusion in BMs’ GTVs have been reported. However, in general, CT/MRI fusion can improve the determination accuracy of GTV in BMs, allowing for personalized treatment options, organ preservation, or functional avoidance. Moreover, it facilitates intensification and dose escalation strategies (**Figure 5**).

## 4 The future direction of GTV determination in BMs

### 4.1 AI-based detection of BMs and automatic segmentation of GTVs

AI is trained and validated on large datasets to provide automated tools to assist physicians in accurately and quickly detecting BMs in large-scale medical imaging datasets. Manually determining BMs’ GTVs is time-consuming and challenging, and autosegmentation significantly enhances efficiency and precision. Deep learning models, such as convolutional neural networks (CNN), have shown promising results in BM image segmentation. Since 2018, AI-based contouring has evolved from classical machine learning (cML) to deep learning (DL). Cho et al. (48) conducted a systematic review of the literature on BM detection based on machine learning. The detection rates of BMs in the cML and DL groups were 88.7% and 90.1%, respectively. The DL group had a lower false-positive rate per patient than the cML group (10 vs. 135), indicating a clear advantage for DL. Deep learning models are adaptable and resilient in handling complex lesion shapes and indistinct boundaries through substantial labeled image data assimilation.

CNN-based AI has gained widespread acceptance for the screening and identification of BMs. Grovik et al. (49) conducted a study evaluating a CNN deep learning method for the automatic detection and segmentation of BMs using multisequence MRI. The results showed an average sensitivity of 83% for detecting BMs, indicating remarkable accuracy. However, the network’s ability to detect BMs was related to lesion size. It was shown that by using the optimal probability threshold (average sensitivity = 83%), the network showed an average false-positive rate of 8.3 (no size limit) and 3.4 (10 mm^3^ size limit) lesions per case, with the highest sensitivity and lowest numbers of false positives in patients with few metastases.

Zhou et al. (50) developed a DL single-shot detector (SSD) algorithm based on CE T1WI to detect BMs. For the test group, the sensitivity of the baseline SSD was 81%; the sensitivity was 98% for metastases ≥ 6 mm in diameter. The combined algorithm of feature fusion (FF) and SSD developed by Amemiya et al. (51) revealed that the FF and baseline SSDs showed an overall sensitivity of 86.0% and 83.8% and a positive predictive value (PPV) of 46.8% and 45.2%, respectively. Thus, FF SSD significantly improved the small lesion detection without reducing the overall PPV. Li et al. (52) introduced a two-stage deep learning model designed for automatic BM detection and segmentation, achieving a detection sensitivity rate of 91%.

With more data availability and technological advancements, AI is expected to become a crucial tool for diagnosing and treating BMs. However, there are still challenges in brain tumor imaging research related to AI technology. Modeling requires large datasets from multiple centers. However, subjectivity can be introduced during the preprocessing stage when physicians manually segment the images. This necessitates high requirements for consistency in image data quality. Therefore, most AI models for BMs are still in the research stage, and their clinical application requires thorough validation.

### 4.2 Functional MRI in GTV determination of BMs

With the development of functional imaging, biology-guided RT has gradually become part of clinical practice. Biologic target volumes refer to regions within the target volume with varying radiosensitivities, determined by various tumor biological factors, including hypoxia, blood supply, proliferation, apoptosis, cell cycle regulation, infiltration, and metastatic properties. Tumor hypoxia is common in RT due to abnormalities in the tumor vasculature. Hypoxic cells are highly resistant to RT, resulting in a relative lack of local tumor doses. Therefore, identifying and quantifying tumor hypoxia is crucial for improving the effectiveness of RT.

Functional MRI, including dynamic contrast-enhanced MRI (DCE-MRI) and dynamic susceptibility contrast-perfusion-weighted imaging (DSC-PWI), can identify, quantify, and spatially map areas of hypoxia before treatment and track hypoxia changes during radiation (53, 54). It can help improve the radiation dose to the hypoxic RT-resistance area through dose engraving and protect the organ at risk (OAR), and it should be incorporated into the practice of radiotherapy in BMs (55, 56).

Functional MR imaging techniques, such as PWI, diffusion-weighted imaging (DWI), and magnetic resonance spectroscopy, provide clinical insight into tumor metabolism, pathophysiology, and microcirculatory status, and they are increasingly used for GTV determination in BMs.

#### 4.2.1 PWI

PWI can be categorized into two main types: those that use Gd contrast agents and those that do not. DSC and DCE, both based on Gd injections, efficiently evaluate vascular infiltration and neovascularization in the enhanced regions of BMs. DSC quantifies tumor vascular supply and is the most frequently employed PWI technique for brain tumor evaluation (57).

Arterial spin labeling (ASL) is a noninvasive method for examining blood flow changes in BMs without the use of paramagnetic contrast agents and without being influenced by the BBB. This imaging technique uses hydrogen protons present in arterial blood as endogenous tracers to provide accurate results. Soni et al. (58) conducted a quantitative comparison of perfusion values acquired through ASL and DSC in brain tumors, demonstrating a favorable degree of agreement. As a result, ASL, as a noninvasive test, exhibits greater potential for broad utilization.

**Figure 6:**
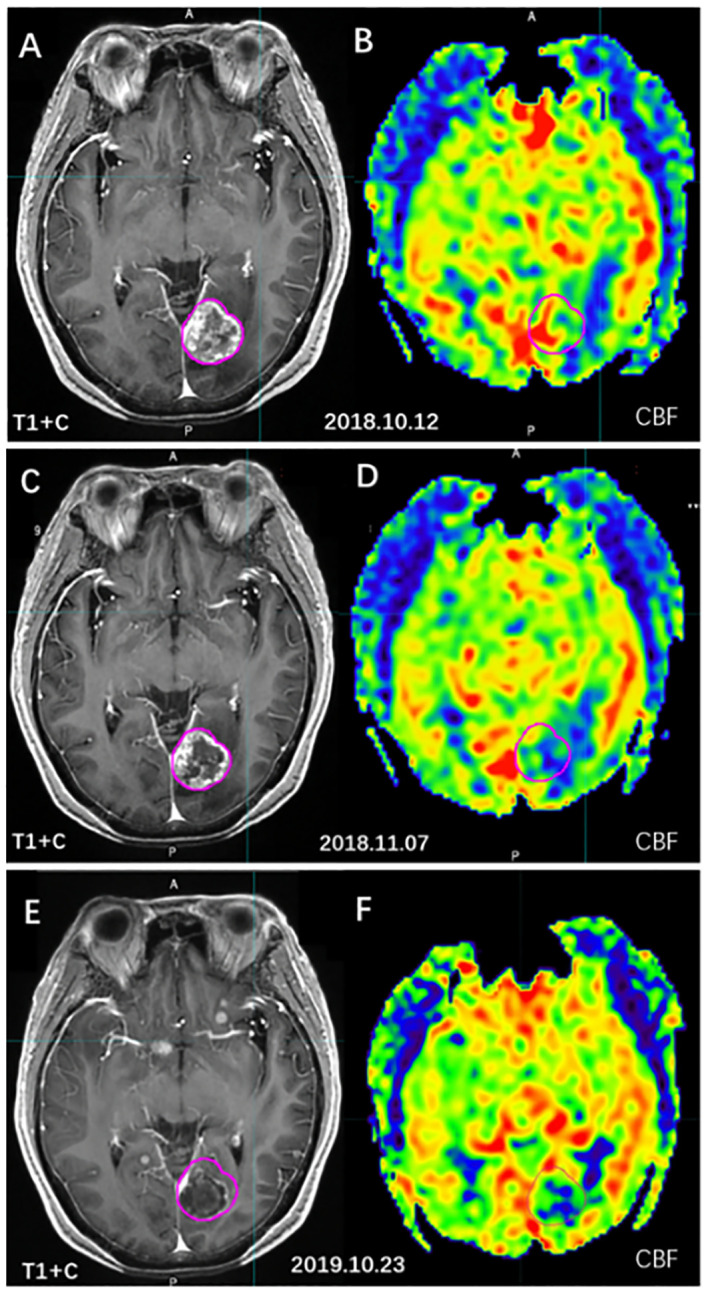
The cerebral blood volume variation of BMs before and after radiotherapy. [**(A, C, E)** T1WI+C images; **(B, D, F)** 3D-ASL cerebral blood volume images; **(A, B)** before radiotherapy; **(C, D)** 4 weeks after radiotherapy; **(E, F)** 1 year after radiotherapy].

Hou et al. (54) used MR-3D-ASL to map cerebral blood flow to determine the high-perfused GTV (GTV_H_) versus the low-perfused GTV (GTV_L_). Their findings revealed an uneven distribution of perfusion within BMs and variations in intratumoral and intertumoral perfusion between tumors with and without necrosis. Dose escalation to low-perfusion areas with RT resistance should be performed by reducing the dose boost volume so that the dose to the target area is targeted and distributed according to blood perfusion. Hou et al. (59) further designed three RT plans based on CBF measurements. When compared to the conventional plan, the dose painting plan exhibited that the D_2%_, D_98%_ (doses to 2% and 98% volume of the PTV), and the mean dose increased by 20.50%, 19.32%, and 19.60% in the low-perfusion region, respectively. Therefore, 3D-ASL-guided dose painting effectively increases the radiation dose to the low-perfusion subregion without increasing the dose to the OAR, and subregion identification and segmentation based on the differences in blood perfusion in the GTV are of great clinical significance (**Figure 6**).

#### 4.2.2 DWI

Conventional MRI sequences have limitations in distinguishing between infiltrating tumors and vasogenic edema. Apparent diffusion coefficient (ADC) values can measure the extent to which water molecules are restricted in healthy brain tissue and central nervous system lesions. On high b-valued DWI, BMs appear as high-intensity signals with low ADC values, which can distinguish tumor and edema.

A study conducted by Zhong et al. (60) identified differences in the ADC values between patients with and without BMs. An ADC value of 0.837 × 10^−3^ mm/s was found to be critical for distinguishing BMs from nonbrain metastases, yielding a sensitivity of 83.7% and a specificity of 69.2%, resulting in a high BM detection rate. However, DWI has low resolution and is prone to magnetization artifacts, and its clinical application in BMs is still mainly qualitative. The study of GTV determination of BMs based on DWI is still under investigation.

## 5 Conclusion and prospects

Accurate target determination is the primary premise for ensuring the efficacy of local RT for BMs. How to improve the accuracy of GTV determination is one of the key issues in improving the efficacy of SRS/SBRT. With the progression of functional imaging technology and AI, methods such as MRI and PET provide more comprehensive data analysis concerning tumor size, infiltration depth, internal microenvironment, and chemical composition within BMs, which provides a feasible method for further improving the detection of BMs and the precision of GTV determination and provides a broad perspective for the clinical application of individualized RT.

## Author contributions

SD: Writing – original draft. GG: Writing – review & editing. RL: Writing – original draft. KM: Writing – original draft. YY: Writing – review & editing.
